# Gambian human African trypanosomiasis in North West Uganda. Are we on course for the 2020 target?

**DOI:** 10.1371/journal.pntd.0007550

**Published:** 2019-08-14

**Authors:** Richard Selby, Charles Wamboga, Olema Erphas, Albert Mugenyi, Vincent Jamonneau, Charles Waiswa, Steve J. Torr, Michael Lehane

**Affiliations:** 1 Vector Biology Department, Liverpool School of Tropical Medicine, Pembroke Place, Liverpool, United Kingdom; 2 Vector Control Division, Ministry of Health, Wandegeya, Kampala, Uganda; 3 Co-ordinating Office for Control of Trypanosomiasis Uganda, Wandegeya, Kampala, Uganda; 4 UMR 177 Intertryp, Institut de Recherche pour le Développement (IRD), Montpellier, France; Makerere University, UGANDA

## Abstract

In 1994, combined active and passive screening reported 1469 cases from the historic Gambian Human African Trypanosomiasis (gHAT) foci of West Nile, Uganda. Since 2011 systematic active screening has stopped and there has been reliance on passive screening. During 2014, passive screening alone detected just nine cases. In the same year a tsetse control intervention was expanded to cover the main gHAT foci in West Nile to curtail transmission of gHAT contributing to the elimination of gHAT as a public health problem in the area. It is known that sole reliance on passive screening is slow to detect cases and can underestimate the actual true number. We therefore undertook an active screening programme designed to test the efficacy of these interventions against gHAT transmission and clarify disease status. Screening was conducted in 28 randomly selected villages throughout the study area, aiming to sample all residents. Whole blood from 10,963 participants was analysed using CATT and 97 CATT suspects (0.9%) were evaluated with microscopy and trypanolysis. No confirmed cases were found providing evidence that the gHAT prevention programmes in West Nile have been effective. Results confirm gHAT prevalence in the study area of West Nile is below the elimination threshold (1 new case / 10,000 population), making elimination on course across this study area if status is maintained. The findings of this study can be used to guide future HAT and tsetse management in other gHAT foci, where reduced caseloads necessitate a shift from active to passive screening.

## Author summary

The number of gHAT cases across West Nile, Uganda has declined in the last 20 years. This decline is due to the impact of programmes of active and passive case detection and treatment which have recently been combined with tsetse control operations (post 2011). We carried out an active survey of gHAT to evaluate the prevalence in areas where vector control has been introduced. Our results confirm that the overall prevalence of gHAT is below 1 case per 10,000 people at risk in the historical foci and shows that results from passive screening are providing an accurate picture of gHAT prevalence in the area.

## Introduction

Human African Trypanosomiasis (HAT) is caused by species of *Trypanosoma brucei* s.l. transmitted by tsetse flies, with case fatality rates believed to be close to 100% [1, 2]. In East and Southern Africa *Trypanosoma brucei rhodesiense* causes Rhodesian HAT (rHAT), an acute and zoonotic form of the disease. In Central and West Africa *Trypanosoma brucei gambiense* causes Gambian HAT (gHAT), the chronic form of disease and is generally considered an anthroponosis [3, 4]. gHAT has been targeted for elimination globally by reducing incidence to less than 1 new case per 10,000 people at risk in 90% of foci, alongside a global total burden of less than 2000 cases by 2020 [5]. In 2017, one of the metrics to assess elimination as a public health problem was revised as ‘total area at risk reporting ≥ 1 case per 10,000 people per year’ [6]. The only country where both forms of the disease occur is Uganda with gHAT present in the north west [7] and rHAT in central and south-eastern parts of the country [8]. Unregulated movement of untreated cattle risk rHAT extending its distribution [9]. Control of gHAT classically relied upon medical surveillance involving either active or passive screening of the population followed by treatment of detected cases. In practice, when prevalence decreases, exhaustive active screening by mobile teams is progressively replaced by passive screening with diagnosis of patients presenting at local health centres [10]. Prompt and correct diagnosis of HAT requires specialist diagnostics, facilities and skilled staff which are frequently not present at most health centres in remote rural areas. Consequently, misdiagnosis and under-reporting of HAT cases by passive screening is an acknowledged problem [11–14].

In Uganda, the annual number of reported gHAT cases declined from 2066 in 1990 to 1469 cases in 1994 and on to just nine cases in 2014 [15]. The driver behind this decline being the large active and passive screening programme by Ugandan National Sleeping Sickness Programme and Médecins Sans Frontières (MSF) France between 1987 and 2002 [16]. As case numbers declined surveillance shifted from large-scale active screening to passive screening. It is plausible that the current low case numbers could be an underestimate due to less effective screening. The last large-scale active screening in West Nile was undertaken by MSF Spain and the Ministry of Health (MoH) supported by WHO in 2010 and 2011. A total of 74,254 people were screened with 22 people diagnosed positive and treated for gHAT at an overall prevalence of 0.029% [17]. All parasitological confirmed cases reported since then have been detected at MoH facilities by passive screening only.

As a contribution to eliminate gHAT as a public health problem in Uganda, tsetse control operations have gradually been expanding in West Nile since 2011. This involves the deployment of insecticide treated Tiny Targets [18] throughout the traditional foci of gHAT managed by the Co-ordinating Office for the Control of Trypanosomiasis in Uganda (COCTU) supported by Liverpool School of Tropical Medicine (LSTM). To evaluate the status of gHAT in areas where Tiny Targets have been deployed, an active screening survey for gHAT has been conducted in the historical gHAT foci of Arua, Maracha, Koboko and Yumbe. Areas of Arua and Maracha have had the Tiny Target tsetse control project since 2011, while in Koboko and Yumbe Tiny Targets were introduced in 2014.

## Materials and methods

### Ethical statement

Sampling activities were conducted in accordance with the Helsinki Declaration. This study was designed in collaboration between LSTM, COCTU, and the National Sleeping Sickness Control Program, Ministry of Health (MoH). Ugandan National Council of Science and Technology granted formal ethical approval (protocol number: HS1749). All samples were collected using the existing national framework for trypanosomiasis screening, employing laboratory technicians from Omugo Health Centre (one of the principal gHAT diagnostic facilities in West Nile region). Care was taken to minimise changes in the techniques used for this study from the standard operating procedure already familiar to the health team.

### Description of study area

This study investigated the historical gHAT foci situated north east of Arua town. Between 2000 and 2012 the study site generated 1448 gHAT cases out of the national total of 3940. The foci extend from 3°06’17.32” to 3°37’10.53” latitude, and 30°56’23.28” to 31°25’57.65” longitude and encompasses areas of Arua, Maracha, Koboko and Yumbe districts. This study area hosts vector control operations through deployment of Tiny Targets. In Arua and Maracha districts control started at scale in 2012 followed by Koboko and Yumbe in late 2014 [18]. HAT cases reported to WHO from each of these areas during the period 2000–2012 were 689 in Arua and Maracha and 759 from Koboko and Yumbe. These two regions form the sub areas of this survey. The population is largely rural, practising small-scale crop farming focussed on food and tobacco. The rivers in the study area include the Enyau, Kochi and Ore along with their smaller tributaries. These rivers support the typical riverine habitat suitable for *Glossina fuscipes fuscipes*, the principal *T*. *b*. *gambiense* vector in this area [18, 19].

### Human medical survey

#### Study design

This study aimed to detect any difference in the number of gHAT cases from areas where vector control had been previously established (Arua and Maracha) and were the technology had very recently become introduced (Koboko and Yumbe). The study is designed as a single stage cluster sampling operated within the defined study areas. Sample site villages within the gHAT focus were selected at random and all residents of selected village clusters were invited for inclusion with an aim to sample each person. To calculate the number of village clusters required, village population figures were obtained from local government officials holding the position of sub-county leader (the information was not available through centralised governmental organisations). Villages had an average of 510 people (range: 217 to 917). Prevalence data from active gHAT screening by MSF in 2010 to 2011 was used to calculate the sample size required in each of the two areas, outlined as follows. Prevalence in Arua and Maracha districts was 0.036% (12/30,726). Koboko and Yumbe was 0.023% (10/43,528). Sample size calculations were completed using Winpepi [20] establishing the number of villages (clusters) needed in both areas. Calculations were made with a confidence interval of 95% and an acceptable difference of 0.08%. Based on this sample size calculation the number of village clusters needed to be sampled was 17 villages between Arua and Maracha (tsetse control introduced at scale in 2011), and 10 villages across Koboko and Yumbe (tsetse control introduced late 2014). Given the average village population is 510 people the anticipated population of 27 sampled villages was 13,770.

Selection of sampling villages utilised a government collated list of Ugandan villages (compiled by Ugandan Bureau of Statistics during 2010). This list was filtered to include all villages within the gHAT focus as defined by WHO derived data on HAT cases since 2005. This generated data of all known villages located in parishes that had been declared as the home of gHAT cases. The list for Arua and Maracha comprised of 449 villages, while the list for Koboko and Yumbe produced 443 villages. Final selection was made by assigning each village a number in numeric sequence and used the random number generator of WinPepi to select numbers within the range of the village list. This process was done twice for the respective sub-areas. After selection of the survey villages, a further ten villages were chosen at random (five for each of the two sub-areas ready to be used if severe problems were encountered in surveying the villages originally selected).

#### Sample protocol

**Fig 1:**
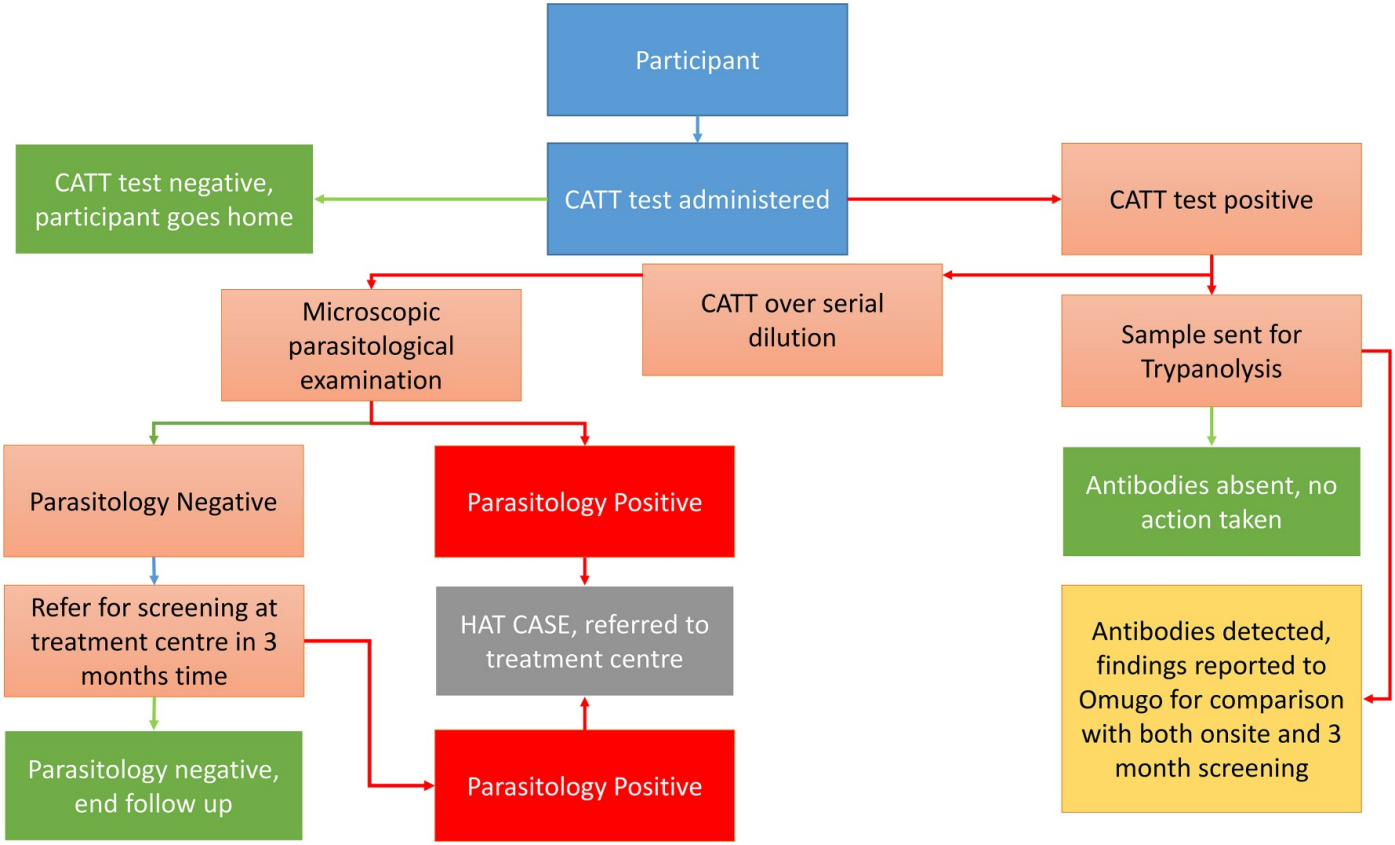
Decision tree for each step of the gHAT sampling, adapted from Simarro et al [21].

Sampling operations ran between June and August 2015. Each village was visited for sensitisation 48 hours before screening in which village chairpersons were informed about the risk posed by gHAT, the method of sampling, that sampling was free and every member of the village was invited to attend. Sampling was carried out between 09:00 and 17:00 h to ensure the team’s safety. Geo-referencing was carried out using Garmin eTrex 20 at each sample site. Participants were given comprehensive details in appropriate languages regarding the sampling procedure and requested to sign an informed consent form. Each participant was then designated a unique identification number to enable tracing of individuals and their age, gender and name of the home village was recorded. Village chairpersons provided the number of residents in their village and worked to identify persons who were from outside of their village. Upon identification, these external participants were still tested but were recorded as not from the village. Fig 1 depicts the diagnostic algorithm used for this survey, indicating the actions for a positive and negative result at each stage of investigation.

Diagnosis of *T*. *b*. *gambiense* was conducted following a three stage protocol consisting of initial screening, parasitological confirmation and finally disease staging of any cases [22]. For initial screening Card Agglutination Trypanosomiasis Tests (CATT) were used. WHO in coordination with the Ugandan National Sleeping Sickness Control Program provided the CATT used in this survey. Each participant was tested using the CATT protocol on whole blood (CATT-WB) [10, 22]. If the CATT-WB test was negative, participants were informed and free to leave. If a test displayed agglutination, samples were advanced to further CATT tests over a series of dilutions; 1:4, 1:8 and 1:16 and microscopic examination took place. Dilution of plasma increases the specificity; agglutination at greater dilution gives an increased suspicion that the participant is positive for gHAT [21, 23]. Participants with a positive CATT-WB result were asked if they had previously had HAT and if so, when (for case history clarification).

Confirmation of gHAT infection was done in accordance with the Ugandan Government MoH guidelines for diagnosis using microscopic examination. The low parasitemia of *T*. *b*. *gambiense* renders unprocessed blood analysis unlikely to find trypanosomes [22]. Following MoH guidelines, we used the Capillary Tube Centrifugation (CTC) technique [24], centrifuging samples at 3000 rpm for five minutes to concentrate trypanosomes within the buffy layer. From each CATT-WB suspect, three capillary tubes were analysed with Leica light microscopes using 40x magnification oil immersion lenses. If suspects (i.e., those with agglutination from CATT) were found microscopically negative during active screening, they were referred for follow up after three months at a regional health centre for final confirmation. If the suspect had palpable cervical lymph nodes a sample was aspirated and examined microscopically.

Each participant with CATT-WB agglutination had a fourth capillary tube of blood collected from the same puncture wound. Blood was applied directly to Whatman filter paper and dried for one hour out of direct sunlight. Samples were then sealed with silica desiccant in individual plastic sachets and stored at 4°C before being dispatched to the WHO collaborating centre in CIRDES laboratory in Bobo Dioulasso, Burkina Faso for analysis by the trypanolysis test [25, 26].

### Cartography

Mapping of the study area was carried out using a combination of open source GIS shapefiles and data from the WHO. National boundaries, rivers and water bodies were obtained from DIVA-GIS [27]. Human population data has been obtained from the Centre for International Earth Science Information Network [28]. Data from the HAT Atlas, provided by WHO, were used to produce a map of the gridded density of HAT cases across the study area. This data was used to show density of gHAT cases based upon reported origin position of each case within cells of a 1km^2^ grid covering the West Nile region. All GIS processing has been conducted using QGIS [29].

## Results

**Fig 2:**
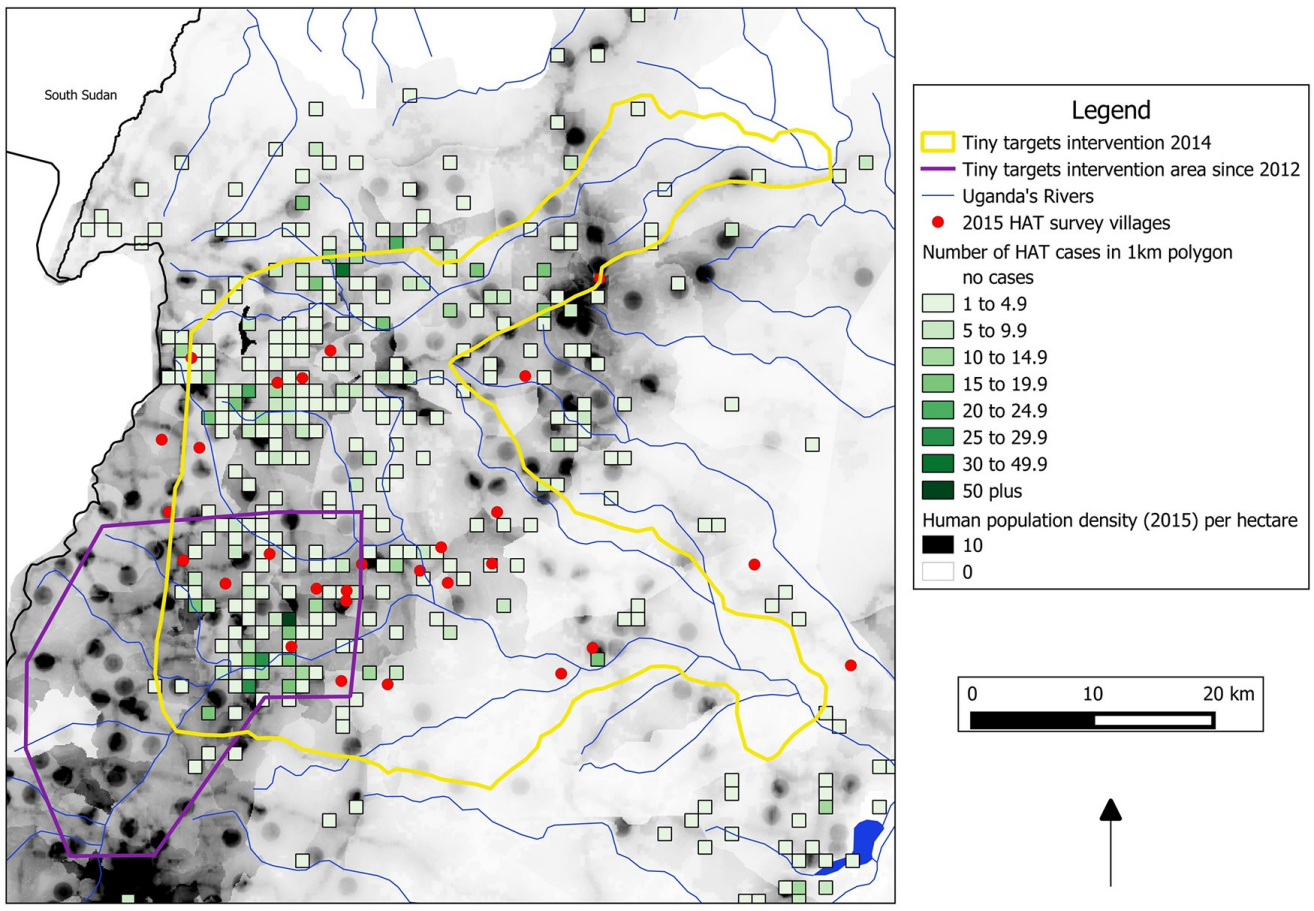
Location of randomly selected sample sites, related to human population density and the origins of past HAT cases.

One of the randomly selected reserve sample sites was added after one original sample site presented a turnout of less than 25% of the population (as provided by village leaders). Therefore, a total of 28 villages were sampled comprising all 27 of the originally selected village sites and a single reserve village. Samples were collected from 10,963 people at 28 village sample sites shown in Fig 2. No sample site was more than 8km from the home village of a gHAT case recorded in the previous decade. The total population of the 28 sample villages was estimated by village leaders as 14,225 inhabitants, giving an overall sample coverage of 77.1%. Of the 10,963 that were screened, 10,500 were residents of the selected sampling villages and 463 were non-residents. Thus, the attendance rate from the selected villages was 73.8%.

### Demographics of the sampled population

**Fig 3:**
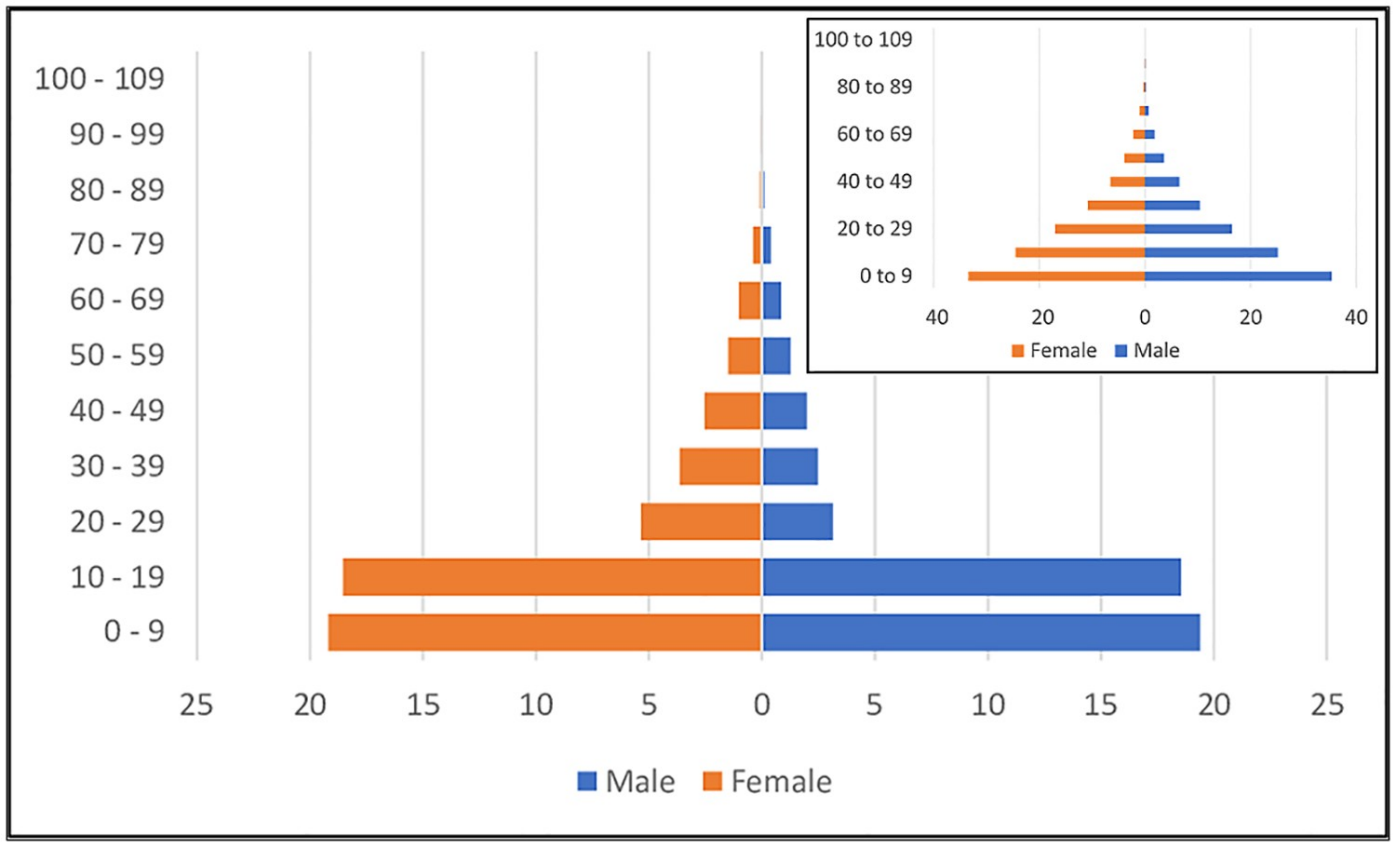
Demographic of the screened population as a percentage of the total screened population (shown on the left, a) and UN derived national demographic data for Uganda (shown on the right, b).

The sampled population comprised 51.9% females and 48.1% males (Fig 3) and the age distribution was in general accordance with the population composition of Uganda, with more young than old. Direct comparison between results from this study and the United Nations’ collated population for Uganda places the UN’s curve from young to old as being far more gradual [30, 31] than our data (Fig 3).

### CATT findings

Initial CATT-WB screening showed agglutination for 0.88% of people screened (97/10963, CI: 0.7–1.1) (Table 1). Following the diagnostic algorithm of these initial positives, 59.2% (61/97, CI: 52.5–72.5) displayed CATT agglutination at 1:4 dilution, 26.2% (27/97, CI: 19.2–37.9) at 1:8 dilution and 13.6% (14/97 CI: 8.1–23.0) at 1:16 dilution. Results of CATT-WB conducted in the sub-areas shows no significant difference (p = 0.8521) between Arua and Maracha (69/7647) and Koboko and Yumbe (28/3316).

**Table 1:** Results of active screening shown by sample (village) site, combined totals included at bottom.

District	Village Name	Total population (reported by village chief)	Villagers sampled	Identified non-villagers	Percent sampled	CATT whole blood	CATT 1:4	CATT 1:8	CATT 1:16	CTC microscopy positive	Trypanolysis positives
**Arua and Maracha**	**CHAKAYI**	874	252	0	28.83	1	1	0	0	0	0
	**EJAVU**	166	165	10	99.40	0	0	0	0	0	0
	**KIDIA**	153	153	23	100.00	1	1	0	0	0	0
	**AMIA**	249	249	13	100.00	1	1	0	0	0	0
	**ODOA**	872	215	0	24.66	3	0	0	0	0	1
	**ILLY HILL**	1028	534	1	51.95	5	4	2	1	0	0
	**MGBAFEA**	704	477	1	67.76	2	1	1	0	0	0
	**ARUMAZE**	619	619	9	100.00	3	2	0	0	0	0
	**AZAAPI**	495	495	214	100.00	4	3	3	2	0	0
	**DUKU**	348	301	0	86.49	6	0	0	0	0	1
	**KOMENDAKU**	964	782	4	81.12	7	7	3	2	0	0
	**AYIVU**	495	495	52	100.00	1	0	0	0	0	0
	**JOYIA**	258	234	2	90.70	2	0	0	0	0	0
	**KANGAYI**	469	410	1	87.42	1	1	1	0	0	0
	**ROBU**	669	560	1	83.71	1	1	1	1	0	0
	**BURA OPIDRI**	700	515	3	73.57	1	0	0	0	0	0
	**ANDELIZO**	500	500	16	100.00	6	5	2	1	0	0
	**AJULEPI**	386	338	3	87.56	1	1	1	1	0	0
**Koboko and Yumbe**	**LUSUGO**	389	203	3	52.19	0	0	0	0	0	0
	**LWANGA**	174	174	32	100.00	0	0	0	0	0	0
	**TUKALIRI**	590	438	5	74.24	3	3	0	0	0	0
	**MONDRUGORO**	212	212	2	100.00	0	0	0	0	0	0
	**DEMGBELENGA**	1467	787	0	53.65	0	0	0	0	0	0
	**YAMIRO**	306	299	2	97.71	2	2	1	0	0	0
	**IJIGO**	169	154	0	91.12	2	0	0	0	0	0
	**EWANYATI**	243	213	1	87.65	7	4	2	1	0	0
	**KITOLI**	167	167	19	100.00	1	1	0	0	0	0
	**ANAFIYO**	559	559	46	100.00	6	5	2	1	0	0
**Cluster village population total**		**14,225**	**10,500**	**463**	**73.81**	**67**	**43**	**19**	**10**	**0**	**2**
**Non-residents of cluster**		**N/A**	**463**	**N/A**	**N/A**	**30**	**18**	**8**	**4**	**0**	**1**
**Combined total**		**14,225**	**10,963**	**N/A**	**77.07**	**97**	**61**	**27**	**14**	**0**	**3**

**Fig 4:**
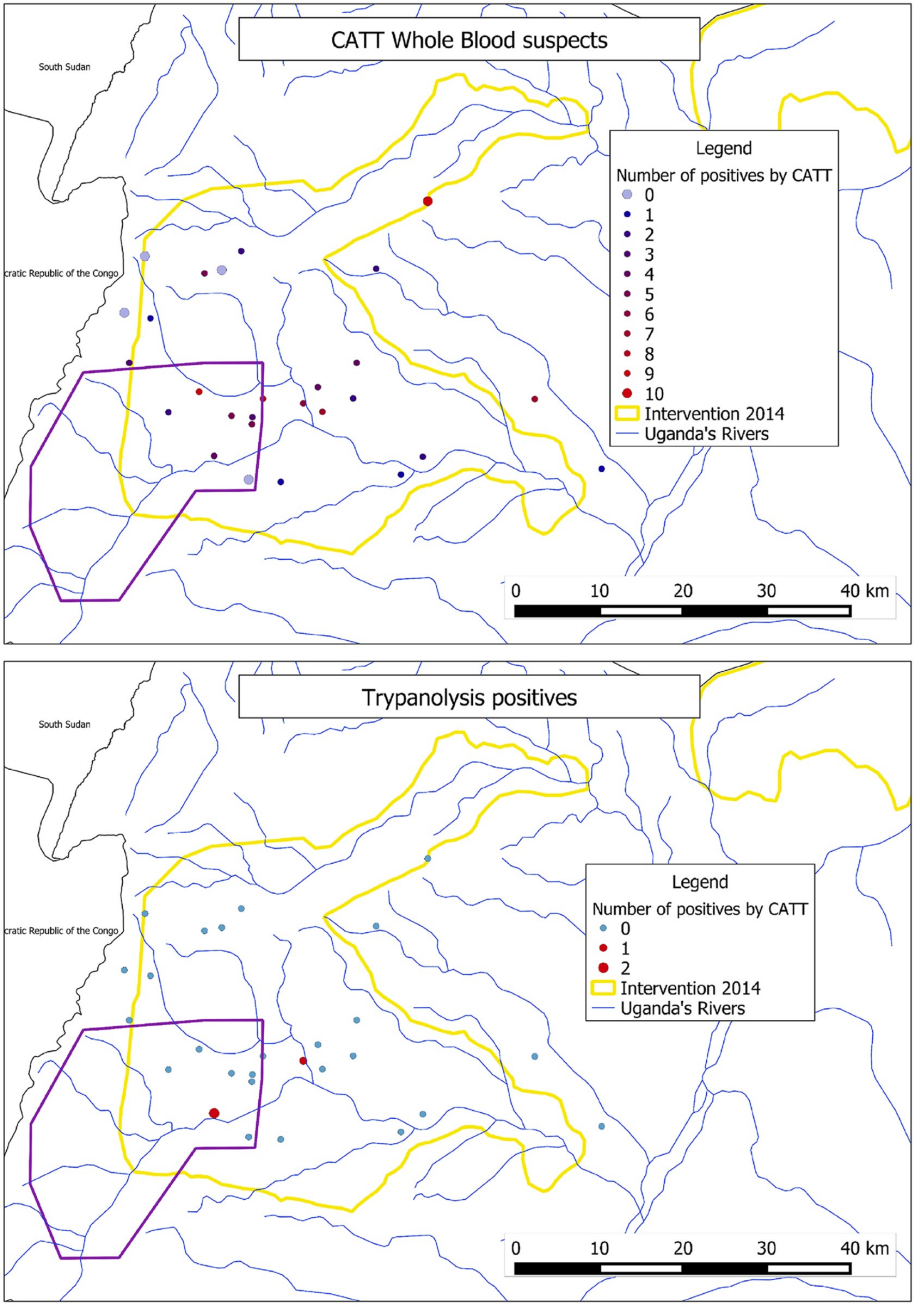
Numbers of CATT on whole blood suspects and number of trypanolysis positives found at each sample point.

Sixty-seven of the 97 CATT-WB suspects were from the selected study cluster villages. The remaining 30 CATT-WB suspects were from other villages (total number of identified attendees from unselected villages was 463). From selected sample villages, 0.64% were CATT-WB suspects (67/10500 CI: 0.54–0.73). CATT-WB suspects from non-selected villages represent 6.48% (CI: 4.4–9.1). Number of CATT-WB per sample site is shown in Fig 4.

### Microscopy

None of the screened participants that were serologically positive by CATT on whole blood (0/97, CI: 0–3.7) were confirmed positive using CTC microscopic analysis to identify trypanosomes. Only one CATT-WB suspect had palpable lymph nodes (lymph was examined microscopically and found negative). All CATT-WB suspects were given appointments for follow up screening after three months and 79 of the 97 suspects attended (76.6%), with none confirmed positive by microscopy. As these individuals had previously been identified as CATT suspects, they were not re-examined by CATT during follow up. As there were no cases diagnosed by microscopy it is not possible to compare the two sub areas of this study.

### Trypanolysis

Analysis of blood indicated that three of 97 undiluted (CI: 0.6–9.0) CATT-WB suspects had previously been infected with *T*. *b*. *gambiense*. The original sampling locations of these three individuals is plotted in Fig 4. All three were from Arua district in neighbouring locations and while it appears from the map that two are from the same village, records show one was from a neighbouring non-selected village.

One trypanolysis suspect had agglutination registered for all dilutions of CATT (1:4, 1:8, 1:16). Two only had agglutination on the whole blood test, no agglutination in dilutions. None had palpable lymph nodes. The individual with consistent agglutination at all dilutions is a past HAT patient who was treated 15 years previously. Geographic assessment of home village locations of the three trypanolysis positive suspects shows they were within 0.5km of the Enyau river system, a documented tsetse habitat [18]. There is no significant difference in the findings of trypanolysis between the sub areas (p = 0.5545), 3/66 from Arua and Maracha and 0/28 from Koboko and Yumbe.

### Past HAT case history

Five of the CATT-WB suspects have previously been HAT cases and while all had received treatment, their staging status is unclear from records. Four were from selected survey villages and one had come from an un-selected village. WHO’s HAT case data from 2000 and 2012 shows the same villages sampled in this study had produced 18 HAT cases. Four of the 18 (22.2%) were identified and resampled during this screening. Given the area that village clusters were selected from has reported 1448 gHAT cases in 892 villages, there was 1430 cases reported to the WHO from the 864 unselected villages.

## Discussion

The absence of confirmed cases in this active screening study supports the conclusion from previous passive screening in West Nile that gHAT transmission in the area is now at a very low level and within the threshold for achieving elimination. The lack of cases makes statistical comparisons of the sub areas with different dates of vector control inappropriate. As a result, the findings are discussed as a single area.

Sampling achieved an average of 73.8% coverage of village populations, comparable to similar surveys [32, 33]. Eleven villages have a sample coverage of over 100%, highlighting the reality of unreliable population estimates compounded by people from outside the sample site attending for screening. As an example, Azaapi sample site had attendance by 214 people from outside the cluster village. This was driven by Azaapi holding both a school and large church resulting in information about sampling spreading to neighbouring villages. In 2015 the Ugandan population was estimated as having much more gradual gradient change from the young to the elderly compared to our data (Fig 3) suggesting our sample may have a disproportionate number of children [30]. As such there was a higher than expected attendance by children which can be attributed to youngsters being encouraged to attend by guardians or school leaders. Data shows a lower number of males reporting for sampling than females, especially between the ages of 20 and 59. This male population may be at a relatively high risk of HAT infection due to increased exposure to tsetse during their working day. Working commitments could also explain their abstention from screening. Partial absence of working age males has been noted in similar medical surveys [34]. Fears inherent in the population have mostly been negated through sensitisation and understanding [32]. In our study extensive sensitisation efforts were made prior to the sampling to encourage population attendance, which exceeded 70%, highlighting its value.

A higher proportion of people from outside selected villages were identified by CATT-WB than those from selected sample cluster villages. Possible contributing factors to this result are people being aware of exposure to tsetse bites or previously been diagnosed as a historical HAT case thereby willing to attend a diagnostic sampling or clinic for their own peace of mind. Investigation into the home villages of these participants shows they were all from within the overall study area centred around historic gHAT foci with the majority coming from the villages immediately neighbouring the randomly selected sample site. This difference in CATT-WB agglutination between the subgroups of the sample suggests that given opportunity, people at high risk of HAT exposure are likely to seek out screening. This may be a reflection of the high levels of awareness of gHAT in these communities [19].

Microscopic examination of the 97 CATT-WB suspects yielded no confirmation of HAT cases during active screening or follow up. Trypanolysis analysis confirmed three suspects as having immunological evidence of *T*. *b*. *gambiense* infection. These three individuals were all residing near one another. Home locations are all within 500m of a known tsetse habitat, placing them at high risk of being bitten by tsetse that could potentially be infected with *T*. *b*. *gambiense*. One of the two immunologic positive individuals that were only identified by CATT on whole blood (i.e., with no agglutination generated from serial dilution) had no HAT case history. As this 27-year-old male has no HAT case history it is possible he is an untreated survivor of the disease and either a potential reservoir or a spontaneous cure [25], meriting follow-ups in the future. The second trypanolysis suspect [a 70-year-old male] only had agglutination on CATT whole blood test with no agglutination on dilution. This individual had been diagnosed and treated for gHAT ten years earlier. The third trypanolysis positive [a 50-year-old female] had agglutination through all dilution ratios despite 15 years having elapsed since treatment for HAT. None of these three individuals was found positive by microscopy during follow up at Omugo health centre. This supports existing work indicating the potential weaknesses in relying solely on CTC microscopy which can miss low parasitaemias [35], particularly in seemingly healthy carriers. These immunologic findings in the absence of confirmation by microscopic analysis of blood could be self-cures or healthy carriers with very low parasitemia in the blood and lymphatic system, undetected by CTC microscopy. Another hypothesis is that the individual has trypanosomes persisting in skin or fat deposits and therefore presents as an immunologic suspect but microscopic negative [36, 37]. These parasites have the potential to remain viable and conceivably form a reservoir for transmission to feeding tsetse.

### Implications to gHAT control policy

Reduction of HAT cases in West Nile has been driven by intensive active and passive medical screening and treatment of infected people between the years of 1987 and 2002 [16]. Recently, as case numbers have dropped, active screening has been scaled back in conjunction with reinforcement of passive screening. This shift has been accompanied by establishment of an effective tsetse control programme which has reduced tsetse apparent densities by over 80% [18]. Our results indicate that the previous combined active and passive screening strategy had a successful impact to reduce HAT cases, which has recently been maintained through strategic passive screening. While tsetse control has not produced the low prevalence of gHAT observed, it will have reduced the risk of undiagnosed infected individuals being fed upon and parasite becoming transmitted [38]. While the sampling turnout for this study (~72%) is considered satisfactory, it highlights the difficulties and risk of relying only on screening and treatment for controlling HAT. This is particularly pertinent in less accessible areas where repeated team visits are impossible, leaving many individuals unscreened. The study was focussed on one of the most active foci of gHAT in Uganda and findings have relevance to other foci since all Ugandan gHAT foci are now benefiting from combined reinforced passive screening and tsetse control.

Since this study concluded, new cases of gHAT from Uganda have remained low; 4 cases in 2015, 4 in 2016 and 2 in 2017 (both of which were in South Sudanese refugees and not Ugandan nationals). Deterioration of security in South Sudan has resulted in an influx of >1 million refugees entering West Nile [39]. This is highly relevant to this study and the Ugandan HAT situation. While effort has been invested and gains made against HAT in Uganda, the WHO HAT atlas [40, 41] shows that there are foci of gHAT in neighbouring areas of South Sudan [15]. Large numbers of people have fled these areas and have settled in refugee camps in various districts of West Nile. Consequently, there is urgent need for raising awareness about HAT in the refugee population and screening for gHAT in refugee camps. Alongside this is a necessity for tsetse control, which focusses on tsetse habitat around the camps and protects the South Sudanese refugees and Ugandan communities from re-emergence of gHAT.

This study demonstrates that the transition from active screening to enhanced passive screening has been successful in providing an accurate representation of gHAT prevalence. The authors therefore recommend that enhanced passive screening should form the basis for control in areas where prevalence is low (be that naturally or having been reduced by active screening operations). In regions of high prevalence active screening still has an important role but should be replaced by enhanced passive screening (as used in West Nile [42]) once a low prevalence has been achieved. As an example, when active screening operations find fewer than five cases per 10,000 people screened, priority can shift to reinforced passive screening combined with focussed active screening at home locations of new cases. Specific thresholds for prioritising reinforced passive screening should be decided by the national MoH with guidance from WHO. This will reduce the burden of cost on the healthcare system.

Alongside screening programmes recent advances in vector control and chemotherapy show promising contributions to gHAT control [42]. Considerations should be taken to implement their usage in future operations as outlined below.

In Uganda, gHAT elimination as a public health problem was attained on the back of the work done prior to the introduction of large-scale vector control. No cases have occurred in areas where Tiny Targets have been deployed. This provides early indication that vector control has helped to maintain incidence at a very low level through directly interrupting transmission. Tsetse control provides a necessary means of continuing the suppression of HAT transmission [38, 43] by minimising the risk posed by infected individuals who miss medical screening and could pass on infection. The consistent stand out risk group in this and other studies is the working age males who represent low attendance figures in screening operations. In addition, tsetse control is especially pertinent if trypanosomes can survive undetected, for example in the skin and fat deposits remaining accessible to feeding tsetse, but are elusive to detection by microscopic analysis. Vector control will also negate any risk posed by potentially viable animal reservoir infections of *T*. *b*. *gambiense* [44].

Advances in orally-administered drugs for gHAT are highly promising since they offer the prospect of an easier and safer treatment for HAT cases with a simpler treatment regimen [45] thereby reducing the burden on health care systems. Treatment options such as nifurtimox-eflornithine combination therapy remain valuable particularly as second line treatment against second stage gHAT. Fexinidazole has been proven capable of diffusing outside of blood and lymph system, into both fat and skin [46]. This presents exciting scope for eliminating transmission from any potential asymptomatic cases through treatment of highly reactive serological or trypanolysis suspects (providing test results are relayed from the lab to local health teams to follow up).

Mathematical models [38, 47] predict that combination of tsetse suppression with medical screening intervention is needed to ensure that WHO’s target of eliminating gHAT as a public health problem (i.e., < 1 case per 10,000 people at risk by 2020) is achieved globally. In Uganda, the health care structure is well developed aiding the success of passive surveillance [48] which permitted the possibility of effective control based largely on active and passive screening. The strategy of reducing incidence through vector control will be important as an accompaniment to medical surveys [43], particularly in regions of gHAT endemicity that lack health centres capable of accomplishing gHAT passive screening.

## Supporting information

S1 Checklist STROBE checklist. (PDF)
