# Supplementary material for: Gambian human African trypanosomiasis in North West Uganda. Are we on course for the 2020 target?
Source: PLoS Negl Trop Dis. 2019 Aug 14;13(8):e0007550. doi: 10.1371/journal.pntd.0007550 (PMC6693741; doi:10.1371/journal.pntd.0007550)
Supplement: S1 Checklist — (PDF) [file pntd.0007550.s001.pdf]

STROBE Statement—Checklist of items that should be included in reports of ***cross-sectional studies***

	Item No	Recommendation
**Title and abstract**	1	(*a*) Indicate the study's design with a commonly used term in the title or the abstract<br>*The study's design is indicated in the abstract*
		(*b*) Provide in the abstract an informative and balanced summary of what was done and what was found<br>*The abstract describes the methods and findings*
**Introduction**		
Background/rationale	2	Explain the scientific background and rationale for the investigation being reported<br>*The background and rational are described in the introduction, paragraphs 1&2*
Objectives	3	State specific objectives, including any prespecified hypotheses<br>*Specific objectives are included in the introduction, paragraph 3*
**Methods**		
Study design	4	Present key elements of study design early in the paper<br>*Study design is discussed in the introduction (paragraph 3) and the methods, paragraph 3 & 4*
Setting	5	Describe the setting, locations, and relevant dates, including periods of recruitment, exposure, follow-up, and data collection<br>*The study's setting is described in the materials and methods. Study location in paragraph 2. Dates for the data collection is in paragraph 5. Recruitment in paragraphs 3 – 5 of the materials and methods*
Participants	6	(*a*) Give the eligibility criteria, and the sources and methods of selection of participants<br>*Site selection is described in paragraph 3 of the materials and methods, protocol for informed consent of volunteers is given in paragraph 5 of the materials and methods*
Variables	7	Clearly define all outcomes, exposures, predictors, potential confounders, and effect modifiers. Give diagnostic criteria, if applicable<br>*Diagnostic criteria and outcomes of each point of the diagnostic algorithm are discussed in the sample protocol sub section, paragraphs 5 – 8 of materials and methods*
Data sources/ measurement	8*	For each variable of interest, give sources of data and details of methods of assessment (measurement). Describe comparability of assessment methods if there is more than one group<br>*Not applicable*
Bias	9	Describe any efforts to address potential sources of bias<br>*Efforts to limit bias are described in the sample protocol sub section, paragraph 5 of the materials and methods*
Study size	10	Explain how the study size was arrived at<br>*Study size calculations are given in the study design sub section, paragraph 3 of the materials and methods*
Quantitative variables	11	Explain how quantitative variables were handled in the analyses. If applicable, describe which groupings were chosen and why<br>*Age of volunteers is the only quantitative variable handled, this is described in paragraph 5 of the materials and methods*
Statistical methods	12	(*a*) Describe all statistical methods, including those used to control for confounding
		(*b*) Describe any methods used to examine subgroups and interactions

		None
		(*c*) Explain how missing data were addressed<br>*None*
		(*d*) If applicable, describe analytical methods taking account of sampling strategy<br>*None*
		(*e*) Describe any sensitivity analyses<br>Paragraphs 5 – 8 *of the materials and methods*
**Results**		
Participants	13*	(a) Report numbers of individuals at each stage of study—eg numbers potentially eligible, examined for eligibility, confirmed eligible, included in the study, completing follow-up, and analysed<br>*Data concerning the numbers of volunteers is given in paragraph 1 of the results section*
		(b) Give reasons for non-participation at each stage<br>*Not applicable*
		(c) Consider use of a flow diagram<br>*Not applicable*
Descriptive data	14*	(a) Give characteristics of study participants (eg demographic, clinical, social) and information on exposures and potential confounders<br>*Demographics of the study's volunteer participants is given in paragraph 2 of the results section*
		(b) Indicate number of participants with missing data for each variable of interest<br>*None*
Outcome data	15*	Report numbers of outcome events or summary measures<br>*Results of all CATT tests, microscopy analysis and trypanolysis ate given in paragraphs 3 – 6 of results section*
Main results	16	(*a*) Give unadjusted estimates and, if applicable, confounder-adjusted estimates and their precision (eg, 95% confidence interval). Make clear which confounders were adjusted for and why they were included<br>*Precision as 95% confidence intervals are given for all results in paragraphs 3 - 6 of the results section*
		(*b*) Report category boundaries when continuous variables were categorized<br>*Not applicable*
		(*c*) If relevant, consider translating estimates of relative risk into absolute risk for a meaningful time period<br>*Not applicable*
Other analyses	17	Report other analyses done—eg analyses of subgroups and interactions, and sensitivity analyses<br>*Trypanolysis is discussed in paragraph 6 of the results section*
**Discussion**		
Key results	18	Summarise key results with reference to study objectives<br>*Key results are summarised in the first paragraph of the discussion*
Limitations	19	Discuss limitations of the study, taking into account sources of potential bias or imprecision. Discuss both direction and magnitude of any potential bias<br>*Limitations are discussed in paragraphs 1 and 2 of the discussion.*
Interpretation	20	Give a cautious overall interpretation of results considering objectives, limitations, multiplicity of analyses, results from similar studies, and other relevant evidence

		Results are cautiously interpreted in paragraphs 1 – 7 of the discussion section
Generalisability	21	Discuss the generalisability (external validity) of the study results<br>*The wider context of the study to other areas with low HAT case reports is discussed in paragraphs 3 - 7 of the discussion*
**Other information**		
Funding	22	Give the source of funding and the role of the funders for the present study and, if applicable, for the original study on which the present article is based<br>*Funders are listed in the additional information given on submission, no in the manuscript*

*Give information separately for exposed and unexposed groups.

Note: An Explanation and Elaboration article discusses each checklist item and gives methodological background and published examples of transparent reporting. The STROBE checklist is best used in conjunction with this article (freely available on the Web sites of PLoS Medicine at http://www.plosmedicine.org/, Annals of Internal Medicine at http://www.annals.org/, and Epidemiology at http://www.epidem.com/). Information on the STROBE Initiative is available at www.strobe-statement.org.
